# Restoration of leg length and offset correlates with trochanteric pain syndrome in total hip arthroplasty

**DOI:** 10.1038/s41598-020-62531-9

**Published:** 2020-04-28

**Authors:** Michael Worlicek, Benedikt Messmer, Joachim Grifka, Tobias Renkawitz, Markus Weber

**Affiliations:** 1 University of Regensburg, Department of Trauma Surgery, University Medical Center, Regensburg, Germany; 2 University of Regensburg, Department of Orthopedic Surgery, Asklepios Medical Center, Bad Abbach, Germany

**Keywords:** Anatomy, Medical research

## Abstract

Persistent pain around the greater trochanter is a common complication after total hip arthroplasty. Restoration of biomechanics such as leg length, femoral und acetabular offset is crucial in THA. The purpose of this study was to evaluate postoperative differences of these parameters after THA and to analyze their association to greater trochanteric pain syndrome. Furthermore, we aimed to evaluate the clinical relevance of trochanteric pain syndrome compared to patient reported outcome measures. 3D-CT scans of 90 patients were analyzed after minimalinvasive total hip arthroplasty and leg length, femoral and acetabular offset differences were measured. Clinical evaluation was performed three years after THA regarding the presence of trochanteric pain syndrome and using outcome measures. Furthermore, the patients’ expectation were evaluated. Patients with trochanteric pain syndrome showed a higher absolute discrepancy of combined leg length, femoral and acetabular offset restoration compared to the non-operated contralateral side with 11.8 ± 6.0 mm than patients without symptoms in the trochanteric region with 7.8 ± 5.3 mm (p = 0.01). Patients with an absolute deviation of the combined parameters of more than 5 mm complained more frequently about trochanteric symptoms (29.2%, 19/65) than patients with a biomechanical restoration within 5 mm compared to the non-affected contralateral side (8.0%, 2/25, p = 0.03). Clinical outcome measured three years after THA was significantly lower in patients with trochanteric symptoms than without trochanteric pain (p < 0.03). Similarly, fulfillment of patient expectations as measured by THR-Survey was lower in the patients with trochanteric pain (p < 0.005). An exact combined restoration of leg length, acetabular and femoral offset reduces significantly postoperative trochanteric pain syndrome and improves the clinical outcome of the patients.

## Introduction

Primary total hip arthroplasty (THA) is one of the most frequently performed orthopaedic operations worldwide, with an increasing number of patients due to an increasing expectation of life^1^. Persistent pain located around the greater trochanter is a common complication after THA impairing mobility and quality of life. In literature this is described as trochanteric pain syndrome and defined as disabling pain localized over the lateral aspect of the hip^2,3^. The prevalence of trochanteric pain syndrome is highly variable in literature. Tan *et al*. report a prevalence of of over 50% in patients presenting in the spine clinic. Barrett *et al*. mention a series prevalence of 27.5%. In addition MRI analysis show peritrochanteric lesion in over 30%^4–6^. Following total hip arthroplasty (THA) 4–17% of patients report these symptoms according to previous studies^7–11^. The reasons of trochanteric pain syndrome as reported in previous studies are manifold and include tendinopathy or microtrauma of the gluteus medius and minimus tendons, often without signs of inflammation^12,13^. In literature a female preponderance for Greater trochanteric pain syndrome in patients without THA has been described^12^.

Beside correct component positioning restoration of biomechanics such as leg length, femoral und acetabular offset is crucial in THA. A recent study showed that even leg length and offset differences over 5 mm are associated with altered gait kinematics as measured in gait analysis after THA and thus emphasize the importance of successful biomechanical restoration^14^. Another study showed, that a reduced global femoral offset less than 5 mm, leads to an aggravation in the functional outcome, especially of the abductor muscle strength^15^. This is in accordance with the findings of Spalding *et al*., who found a reduced motion and strength in the abductor muscle after reduced femoral offset reconstruction in THA^16^. However, the correct radiographic evaluation of leg length and offset is challenging since plain radiographs harbor the risk of misinterpretation of these parameters due to the variable position of the pelvis in relation to the plane of the film. Especially assessment of femoral offset on plain radiographs seems to be defective and can be underestimated up to 40%^17^. In contrast three-dimensional computed tomography (3D-CT) enables exact evaluation of leg length and offset with inaccuracies below 1 mm^18^. To the best of the authors’ knowledge no study has analyzed the association of combined leg length and offset restoration and trochanteric pain syndrome after THA so far.

The purpose of this study was to evaluate postoperative leg length, femoral and acetabular offset differences after THA using 3D-CT and to analyze their association to greater trochanteric pain syndrome. Furthermore, we aimed to evaluate the clinical relevance of trochanteric pain syndrome compared to patient reported outcome measures (PROMs).

## Material and methods

This study is a secondary outcome analysis of a registered, prospective controlled trial (DRKS00000739, German Clinical Trials Register)^19^. The primary outcome of this larger study was to assess whether the ROM of the prosthetic joint could be improved by computer-assisted functional optimization of position and containment of the acetabular component. A cohort of 783 patients with hip osteoarthritis was screened. The inclusion criteria of the initial study were: age between 50 and 75 years, an American Society of Anesthesiologists (ASA) score of ≤3, unilateral osteoarthritis of the hip (up to Kellgren 2 of the contralateral side), no prior hip surgery, and no hip dysplasia or trauma. In total, 597 patients did not meet the inclusion criteria. Altogether a consecutive series of 135 patients were enrolled in this single center study.

The current study is a prospective analysis independent from the primary study. The primary study dealt with ROM improvement by computer-assisted, functional optimization of cup position in primary THA. In the current analysis, we focused on the clinical outcome and source of greater trochanteric pain syndrome after THA in relation to restoration of biomechanics such as leg length and offset. A sovereign power calculation was performed for the study endpoint reduction of postoperative greater trochanteric pain syndrome in relation to accuracy of biomechanical restoration. We assumed a reduction of greater trochanteric pain syndrome about 25% if leg length and offset was successfully restored. Based on these considerations, a total sample size of 84 (63/21) with 3 to 1 distribution achieved a power of 80% using a two-tailed t test on a two-sided 5% significance level (GPower 3.1, Düsseldorf, Germany).

**Figure 1:**
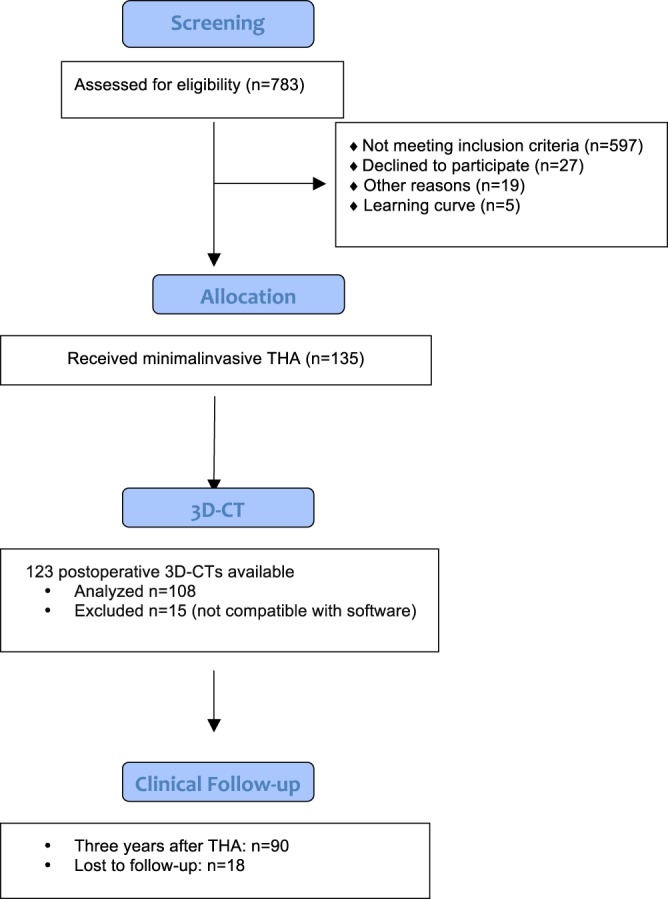
Consolidated Standards for Reporting Trials flow diagram for participants. (THA = total hip arthroplasty).

According to the inclusion and exclusion criteria of the main study 135 patients undergoing minimally-invasive THA were included in the study. Postoperative 3D-CTs for leg length and offset measurements were available for 123 study participants. Out of this 123 data sets 15 CT files were not compatible with the software for 3D-CT measurement of leg length, femoral and acetabular offset. Ninety patients of this subgroup were available for clinical follow up of trochanteric pain syndrome and PROMs three years after THA and thus included in the final analysis (Fig. 1). Characteristics of the study group are shown in Table 1. This investigation was approved by the local Ethics Committee of the University of Regensburg, Germany (No. 10-121-0263). All operations were performed in the lateral decubitus position through a minimally-invasive anterolateral approach to the hip at our Department of Orthopedic Surgery. Preoperative leg length and offset differences were measured on magnification-corrected low-centered anteroposterior pelvic radiographs. The aim during the operation was to restore leg length and offset in relation to the non-affected contralateral side.

**Table 1:** Characteristics of the study group*.

	n = 90
Gender (female) (%)	47 (52.2)
Age (yrs)	62.1 (SD 7.8)
BMI (kg/m^2^)	26.6 (SD 3.9)
ASA 1 (%)	17 (18.9)
ASA 2 (%)	49 (54.4)
ASA 3 (%)	24 (26.7)
Treatment side (right) (%)	52 (57.8)

BMI = body mass index; ASA = American Society of Anaesthesiologists.

**Figure 2:**
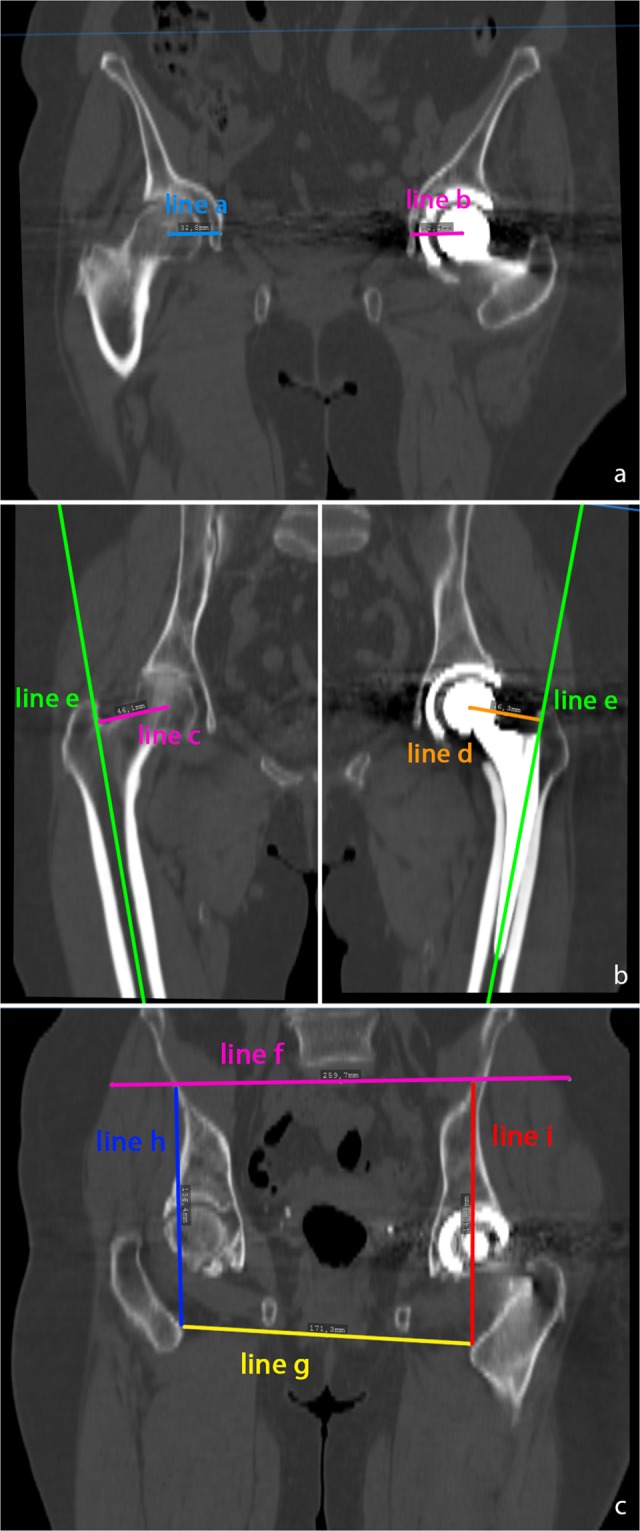
(**a**) Measurement of the acetabular offset in the coronar plane. Acetabular offset was defined as the shortest distance from the center of the femoral head to the bottom of the acetabulum. Line a nonoperated side, line b after THA. (**b**) Measurement of the femoral offset. Femoral offset was defined as the shortest distance from the center of the femoral head perpendicular to the femoral shaft axis. Line c represents the femoral offset on the nonoperated side, line d represents the femoral offset after THA, line e represents the femoral shaft axis. (**c**) Measurement of the leg length difference. Line f represents the height of both anterior superior iliac spines, line g represents both lesser trochanter minors. The difference between line h and i represents the leg length difference.

Postoperatively, 3D-CTs were obtained in all patients and leg length, femoral and acetabular offset differences were measured. We chose 3D-CT for our analysis since measurements especially of femoral offset on plain radiographs are susceptible to error. In contrast, 3D-CT offers the highest accuracy in estimation of leg length and offset independently of the position of the pelvis^17^. CT measurements were carried out using the ‘semi-automatical’ function of a digital 3D-CT-based planning software (Modicas, Erlangen, Germany). This software offers the possibility to assess hips in three dimensions, to exactly determine the axes and to automatically calculate angles and measure distances. First, the pelvis was virtually aligned in order to bring the anterior pelvic plain in congruence with the coronal plain, to have a neutral pelvic tilt and so a constant starting point. In order to exclude any rotational errors both anterior superior iliac spines (ASIS) were orientated to the same height in the coronal, sagittal and axial plane. Acetabular offset was defined as the shortest distance from the center of the femoral head to the bottom of the acetabulum (Fig. 2a), femoral offset was defined as the shortest distance from the center of the femoral head perpendicular to the femoral stem axis (Fig. 2b). Both sides were measured and the difference was built between operated and non-operated side. The nonoperated side was used as reference for the true offset and leg length. Correspondingly, to assess the leg length difference, the distance from the ASIS to the lesser trochanter was measured on both sides and the difference was built (Fig. 2c). For a comparable analysis, the absolute values of all three differences, independent if over- or under-corrected, were added together, to built a combined value of the measured parameters.

Clinical evaluation was performed three years after THA by an independent observer blinded to the radiographic results, regarding the presence of trochanteric pain syndrome. Trochanteric pain syndrome was defined as tenderness on palpation of the greater trochanter and painful active abduction of the hip. Preoperatively and 3 years after THA, outcome measures such as the Harris Hip Score (HHS)^20^, Hip Disability And Osteoarthritis Outcome Score (HOOS)^21^ and EuroQol (EQ-5D)^22^ were obtained. Furthermore, the patients’ expectation were evaluated using the Hospital for Special Surgery Total Hip Replacement Expectations (THR) Survey^23^. Preoperatively, none of the patients had symptoms of a trochanteric pain syndrome as defined before. The HHS first published in 1969 was established as a new method to evaluate the results after mold arthroplasty^15^. Since then it has been widely used for assessing outcome after hip surgery^24^. The HHS covers four dimensions: pain, function, deformity and range of motion. Altogether the HHS consists of 10 items resulting in a maximum score of 100 points. As a clinician based outcome measure it has be obtained by physician or other qualified health care professional. For interpretation score values 100–90 points represent an excellent result, 90–80 points a good result, 80–70 points a fair result and <70 points a poor results, respectively [9]. In contrast to HHS all other outcome measures in this study are patient reported. The HOOS was developed to measure outcome in patients with hip osteoarthritis. All Western Ontario and McMaster Universities Osteoarthritis Index (WOMAC)^25^ questions are included in the HOOS. In addition to WOMAC, HOOS contains subscales for sport and recreation function resulting in a better responsiveness especially in younger patients^26^. The HOOS consists of 5 subscales: pain, symptoms, activities of daily living, sport and quality of life built by 40 items. For standardized answers five Likert-boxes are available. The best scale is 100 points indicating no problems^27^. The EQ-5D is a widely used and tested descriptive instrument for evaluating health. It defines health based on five dimensions: Mobility, Self-Care, Usual Activities, Pain/Discomfort, and Anxiety/Depression. Each dimension has 3 response categories ranging from no problems, some problems to extreme problems. The EQ-5D was tested in general population and patient samples for valuing health^28^.

For statistical analysis, continuous data are presented as mean (standard deviation). Group comparisons were performed by Mann-Whitney-U-tests. Absolute and relative frequencies were given for categorical data and compared between groups by chi-square tests. All hypothesis in the study was tested on 5% significance level. Multivariate logistic regression including gender, age, ASA, BMI and Kellgren was performed to search for risk factors associated trochanteric pain syndrome after THA. IBM SPSS Statistics 22 (SPSS Inc, Chicago, IL, USA) was used for analysis.

### Ethical approval

This investigation was approved by the local Ethics Committee (No.10-121-0263). All procedures were in accordance with the ethical standards of the responsible committee on human experimentation and with the Helsinki Declaration of 1975, as revised in 2000.

### Informed consent

Informed consent was obtained from all individual participants included in the study.

## Results

The prevalence of trochanteric pain syndrome in our cohort was 23.3% (21/90).

**Figure 3:**
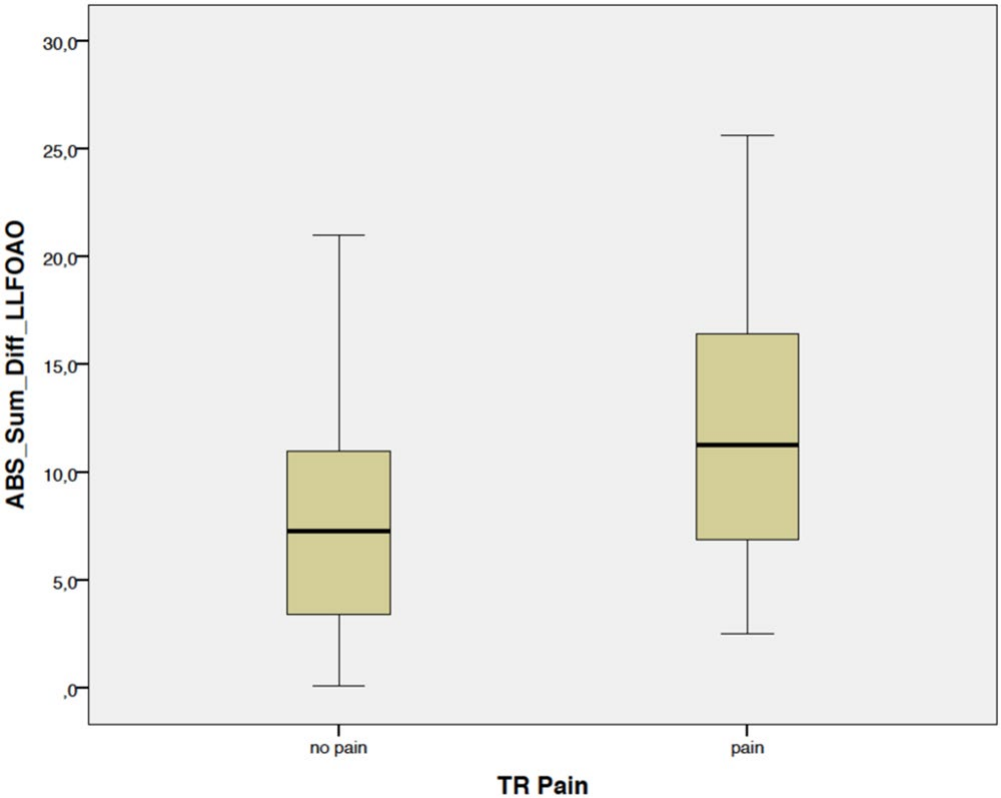
Patients with trochanteric pain syndrome three years after THA showed a higher absolute discrepancy of combined leg length, femoral and acetabular offset restoration compared to the non-operated contralateral side with 11.8 ± 6.0 mm than patients without symptoms in the trochanteric region with 7.8 ± 5.3 mm (p = 0.01). ABS_Sum_Diff_LLFOAO = absolute sum of differences of leg length, femoral and acetabular offset compared to the non-operated side, TR pain = trochanteric pain syndrome.

Patients with trochanteric pain syndrome three years after THA showed a higher absolute discrepancy of combined leg length, femoral and acetabular offset restoration compared to the non-operated contralateral side with 11.8 ± 6.0 mm than patients without symptoms in the trochanteric region with 7.8 ± 5.3 mm (p = 0.01; Fig. 3). Patients with an absolute deviation of combined biomechanical restoration of leg length, femoral and acetabular offset of more than 5 mm complained more frequently about trochanteric symptoms (29.2%, 19/65) than patients with a biomechanical restoration within 5 mm compared to the non-affected contralateral side (8.0%, 2/25, p = 0.03). In contrast, the discrepancy of one single biomechanical parameter such as leg length, femoral or acetabular offset was not different between patients with and without trochanteric symptoms (Table 2).

**Table 2:** Discrepancy (absolute values) of one single biomechanical parameter such as leg length (LL), femoral (FO) or acetabular offset (AO) was not different between patients with and without trochanteric symptoms.

		LL (mm)	FO (mm)	AO (mm)
No pain	mean	4.9	4.4	4.3
	SD	4.3	3.2	3.2
	median	4.2	3.8	3.9
	minimum	0.0	0.1	0.0
	maximum	18.9	15.3	16.4
trochanteric pain syndrome	mean	6.7	5.4	3.8
	SD	5.6	3.7	2.7
	median	5.6	5.2	2.6
	minimum	0.6	0.7	0.8
	maximum	25.4	16.0	10.4
	p-value	0.10	0.26	0.45

SD = Standard Deviation.

Clinical outcome measured three years after THA such as HHS, HOOS and EQ-5D was significantly lower in patients with trochanteric symptoms than in patients without trochanteric pain (p < 0.03, Table 3). Similarly, fulfillment of patient expectations as measured by THR-Survey was lower in the patient with trochanteric pain at each followup point (p < 0.005). In contrast, no differences in clinical socres were observed in the preoperative situation.

**Table 3:** Harris-Hip-Score (HHS), Hip Disability And Osteoarthritis Outcome Score (HOOS), EuroQol (EQ-5D) and Hospital for Special Surgery Total Hip Replacement Expectations (THR) Survey were obtained before and three years after THA according to the development of trochanteric pain syndrome. For quantitative data values are given as mean (SD = standard deviation).

		HHS preop	HHS 3 y postop	HOOS preop	HOOS 3 y postop	EQ-D5 preop	EQ-D5 3 y postop	THR preop	THR 3 y postop
No TPS	mean	50.2	97.0	38.5	90.1	0.6	1.0	84.9	87.1
	SD	10.3	6.7	12.4	11.2	0.3	0.1	14.6	14.8
	minimum	22	57.0	1.9	45.0	0.1	0.7	30.6	37.5
	maximum	67.0	100	62.5	100.0	1.0	1.0	100.0	100.0
TPS	mean	47.9	93.1	38.1	81.4	0.6	0.9	80.9	74.8
	SD	14.4	8.6	15.2	17.3	0.3	0.2	15.3	16.1
	minimum	10.0	64.0	5.0	37.5	0.1	0.1	48.6	28.9
	maximum	64.0	100	64.4	100.0	1.0	1.0	100.0	100
	p-value	0.80	0.006	0.80	0.03	0.55	0.003	0.27	0.001

TPS = trochanteric pain syndrome, preop = preopertavie, postop = postoperative, SD = Standard Deviation.

Multivariate analysis confirmed the independent association of trochanteric pain syndrome and accuracy of combined biomechanical restoration of leg length, femoral and acetabular offset (OR 1.1, 95% CI 1.0–1.3, p = 0.006) whereas other parameters such as gender, age, ASA, BMI and Kellgren score did not correlate with trochanteric symptoms (Table 4).

**Table 4:** Multivariate analysis of risk factors associated with trochanteric pain syndrome. HR = Hazard Rate, CI = Confidence Interval, Combined LL/FO/AO = sum of absolute values of the differences of leg length (LL), femoral offset (FO) and acetabular offset (AO) between operated and nonoperated side.

	HR	95% CI	P-value
Combined LL/FO/AO	1.1	1.0–1.3	<0.01
Gender	0.8	0.3–2.5	0.7
Age	1.0	0.9–1.0	0.4
ASA	1.5	0.6–3.8	0.3
BMI	1.0	0.9–1.1	0.9
Kellgren	0.7	0.4–1.4	0.3

ASA = American Society of Anaesthesiologists, BMI = Body Mass Index.

## Discussion

Trochanteric pain syndrome complicates up to 17% of cases after THA^29,30^ and has a 5- year post-operative incidence of up to 4.9%, depending on the approach^9^. Although this complication can successfully treated with corticosteroid injections^9,11^, Silva *et al*. showed no evidence of bursitis or inflammation histologically after THA in patients with greater trochanteric pain syndrome^31^. Mahmood *et al*. showed, that a reduced global femoral offset less than 5 mm, leads to an aggravation in the functional outcome, especially of the abductor muscle strength^15^. This is in accordance with the findings of Spalding *et al*., who found a reduced motion and strength in the abductor muscle after reduced femoral offset reconstruction in THA^16^. Following these results, we presumed that a deviation of the three-dimensional hip biomechanics from the native situation might correlate with these symptoms after THA.

There are several limitations to our study. First, our results are based on 90 patients with unilateral osteoarthritis of the hip. Therefore, the study is restricted by numbers. However, a power calculation was performed preoperatively according to the posed hypothesis of the study. Second, patients with anatomical disorders such as dysplasia and patients with prior trauma were not included in the study because of the strict inclusion criteria of the main study. Such patients may have a different relation between individual anatomy and greater trochanteric pain syndrome. Third, a minimally-invasive, anterolateral approach to the hip was used in the current study. Previous studies assumed an association between trochanteric pain and the applied surgical approach. Disruption of soft tissue during a lateral approach with resultant abductor tear, tendon defects and tendinitis might be associated with the postoperative prevalence of TPS whereas anterior and posterior approaches might be less prone to TPS. Therefore, the results might be different when using a different surgical approach^9^.

Fourth, there might be different reasons for the development of trochanteric pain syndrome like gait disturbances or lower back problems. We did not analyze these factors particularly, but we have to emphasize that no patient in out cohort had symptoms of a trochanteric pain syndrome before surgery. A strength of our study is its 3D-CT scan dependent measuring protocol of leg length, femoral and acetabular offset, which allowed an exact determination of the anatomy of the hip joint and the used parameters, minimalizing inaccuracy as it occurs on plain radiographs due to projection failures^17^.

In answer to the first question of the study, we found patients with Greater trochanteric pain syndrome three years after THA had a higher absolute discrepancy of combined leg length, femoral and acetabular offset restoration compared to the non-operated contralateral side than patients without symptoms in the trochanteric region. Due to the fact, that we used absolute values, both over- as well as underrestoration of leg length and offset compared to the non-operated side are associated to greater trochanteric pain syndrome after THA. Patients with an absolute deviation of combined biomechanical restoration of leg length, femoral and acetabular offset of more than 5 mm complained approximately three times more frequently about trochanteric symptoms, than patients with a biomechanical restoration within 5 mm compared to the non-affected contralateral side. So there seems to be a tolerance interval for the reconstruction of femoral offset, acetabular offset, and leg length within these 5 mm at least. This is in accordance with two previous studies, which showed, that a reduced global femoral offset less than 5 mm, lead to an aggravation in the functional outcome, especially of the abductor muscle strength and that a leg length and offset difference over 5 mm was associated with altered gait kinematics^14,15^. Interestingly, our results show that the discrepancy of one single biomechanical parameter such as leg length, femoral or acetabular offset was not different between patients with and without trochanteric symptoms. These results are in accordance with the findings of Abdulkarim *et al*., who showed no relationship between greater trochanteric pain syndrome and femoral offset or femoral center of rotation^29^. These results suggest, that the restoration of hip anatomics in THA has to be assessed three-dimensionally^17^.

Furthermore, this study showed that clinical outcome measured three years after THA such as HHS, HOOS and EQ-5D was significantly lower in patients with trochanteric symptoms than patients without trochanteric pain, as well as fulfillment of patient expectations as measured by THR-Survey was lower in the patient with Greater trochanteric pain syndrome. These results emphasize the clinical relevance of Greater trochanteric pain syndrome on outcome and satisfaction of patients after THA. To classify these results, it is necessary to take a look at the general outcome after THA. A recent study, which analyzed 1234 primary total hip replacements, showed excellent outcome for patients after THA with a positive responder rate of 93% and corresponding PROMs using WOMAC and EQ-5D^32^. This is in accordance with different studies, which analyzed the long-term outcome of patients after primary THA^33–35^. The promising results in outcome even apply to patients with severe diseases like haemophilia and rheumathoid arthritis^36,37^.

In addition, the results of Robertson-Watres *et al*. showed a poor outcome for patients with surgical treatment for greater trochanteric pain syndrome after THA, analyzing patient satisfaction score; 10 cm visual analogue score referring to current level of pain (0–100, 0 representing no pain); Oxford hip score; Likert scale duration of pain relief following the operation; Binary presence of pain when lying on affected side^8^. This confirms the importance of accurate three-dimensional restoration of the hip joint biomechanics and shows the necessity to prevent this complication already during the initial surgery.

## Conclusion

In conclusion, combined restoration of leg length, acetabular and femoral offset is crucial in THA. An exact combined restoration of these biomechanical parameters reduces significantly postoperative trochanteric pain syndrome and improves the clinical outcome of the patients. A restoration up to 5 mm seems to be safe for avoiding this complication.
